# Association of triglyceride-glucose index and its related parameters with atherosclerotic cardiovascular disease: evidence from a 15-year follow-up of Kailuan cohort

**DOI:** 10.1186/s12933-024-02290-3

**Published:** 2024-06-19

**Authors:** Xue Xia, Shuohua Chen, Xue Tian, Qin Xu, Yijun Zhang, Xiaoli Zhang, Jing Li, Shouling Wu, Anxin Wang

**Affiliations:** 1 Department of Epidemiology, Beijing Neurosurgical Institute, Beijing Tiantan Hospital, Capital Medical University, Beijing, China; 2 China National Clinical Research Center for Neurological Diseases, Beijing Tiantan Hospital, Capital Medical University, Beijing, China; 3 Department of Cardiology, Kailuan General Hospital, North China University of Science and Technology, Tangshan, Hebei China; 4 Department of Clinical Epidemiology and Clinical Trial, Capital Medical University, Beijing, China; 5 Department of Epidemiology and Health Statistics, School of Public Health, Capital Medical University, Beijing, China; 6 Department of Epidemiology, Beijing Neurosurgical Institute, Beijing Tiantan Hospital, Capital Medical University, No. 119 South Fourth Ring West Road, 100070 Beijing, China

**Keywords:** Triglyceride glucose (TyG), Triglyceride glucose-body mass index (TyG-BMI), Triglyceride glucose-waist circumference (TyG-WC), Triglyceride glucose-waist-height ratio (TyG-WHtR), Atherosclerotic cardiovascular disease (ASCVD)

## Abstract

**Background:**

Triglyceride glucose (TyG) index and its related parameters have been introduced as cost-effective surrogate indicators of insulin resistance, while prospective evidence of their effects on atherosclerotic cardiovascular disease (ASCVD) remained scattered and inconsistent. We aimed to evaluate the association of TyG and its related parameters with new-onset ASCVD, and the predictive capacity were further compared.

**Method:**

A total of 95,342 ASCVD-free participants were enrolled from the Kailuan study. TyG and its related parameters were defined by fasting blood glucose, triglyceride, body mass index (BMI), waist circumstance (WC) and waist-to-height ratio (WHtR). The primary outcome was incident ASCVD, comprising myocardial infarction (MI) and ischemic stroke (IS). Cox proportional hazard models and restricted cubic spline (RCS) analyses were adopted to investigate the association between each index and ASCVD. The C-index, integrated discrimination improvement (IDI), and net reclassification improvement (NRI) were used for comparison of their predictive value for ASCVD.

**Results:**

During a median follow-up of 15.0 years, 8,031 new cases of ASCVD were identified. The incidence rate of ASCVD increased along with elevated levels of each index, and the relationships were found to be nonlinear in the RCS analyses. The hazard ratio (HR) and 95% confidence interval (95% CI) for ASCVD was 1.39 (1.35, 1.43), 1.46 (1.41, 1.50), 1.50 (1.46, 1.55), and 1.52 (1.48, 1.57) per 1 IQR increase of baseline TyG, TyG-BMI, TyG-WC, and TyG-WHtR, respectively, and the association were more pronounced for females and younger individuals aged < 60 years (*P*_for interaction_<0.05). Using the updated mean or time-varying measurements instead of baseline indicators did not significantly alter the primary findings. Additionally, TyG-WC and TyG-WHtR showed better performance in predicting risk of ASCVD than TyG, with the IDI (95% CI) of 0.004 (0.001, 0.004) and 0.004 (0.001, 0.004) and the category-free NRI (95% CI) of 0.120 (0.025, 0.138) and 0.143 (0.032, 0.166), respectively. Similar findings were observed for MI and IS.

**Conclusions:**

Both the TyG index and its related parameters were significantly and positively associated with ASCVD. TyG-WC and TyG-WHtR had better performance in predicting incident ASCVD than TyG, which might be more suitable indices for risk stratification and enhance the primary prevention of ASCVD.

**Supplementary Information:**

The online version contains supplementary material available at 10.1186/s12933-024-02290-3.

## Background

Despite remarkable advance in prevention strategies and therapeutic techniques, the burden of atherosclerotic cardiovascular disease (ASCVD) remains its decades-long rise for almost all non-high-income countries, and has becoming the leading cause of mortality and health impairment worldwide [1]. It was estimated that ASCVD accounted for 22% of total deaths and 245.5 million disability-adjusted life years (DALYs) in 2019, for which metabolic dysfunction was acknowledged as a predominant contributor [2, 3]. Therefore, optimization of existing strategies to identify and control metabolic risk factors is essential for better prevention of ASCVD.

Impaired insulin sensitivity, also known as insulin resistance (IR), contributes to the pathophysiology of diabetes and serves as a crucial element contributing to ASCVD [4]. The triglyceride glucose (TyG), defined merely based on fasting blood glucose (FBG) and triglyceride (TG), has gradually been used as a convenient and reliable alternative index for the consuming and expensive hyperinsulinemic-euglycemic clamp technique, which remains the gold standard for assessing insulin sensitivity [5, 6]. Moreover, several studies revealed the association of TyG index with risks of hypertension, myocardial infarction (MI), and ischemic stroke (IS), some of which further confirmed its clinical value in predicting ASCVD [7–9]. However, no agreement has been reached on whether accounting for IR could improve the risk prediction of ASCVD due to the conflicting findings from previous studies [10]. 

Fat mass and central obesity also play an important role in the progress of atherogenic endothelial dysfunction partly through metabolic disorders, which ultimately lead to the occurrence of ASCVD [11, 12]. Thus, TyG combined with obesity indices like body mass index (BMI), waist circumference (WC) and waist-to-height ratio (WHtR), were introduced as new surrogates for IR and were proved to be associated with a series of metabolic diseases encompassing diabetes mellitus, metabolic syndrome, hypertension, and non-alcoholic fatty liver disease [13–16]. More recently, epidemiological evidence has suggested that those TyG related parameters were closely related to ASCVD and some even showed better prediction ability over TyG, while the evidence was scattered and most of them were drawn from cross-sectional designed studies, leaving the conclusion inconsistent [17–20]. 

With regards to the above, the current analysis was conducted based on the Kailuan cohort to investigate the association of TyG, TyG-BMI, TyG-WC, and TyG-WHtR with ASCVD and its subtypes, using the updated mean and time-varying indicators instead of baseline measurements in the sensitivity analyses for further verification. Additionally, we compared the predictive value of the TyG and its related parameters for ASCVD by calculating C-index, integrated discrimination improvement (IDI), and category-free net reclassification index (NRI).

## Methods

### Study design and participants

The study population was derived from the Kailuan study, a community-based prospective cohort study conducted in Northern China, which has been fully described in previous publications [21–23]. In brief, it was established during 2006–2007, recruiting 101,510 participants aged 18–98 years from the Kailuan company. The subsequent visits were performed every two years until December 2021. The study complied with the Declaration of Helsinki and was approved by the Ethics Committees of both Kailuan General Hospital and Beijing Tiantan Hospital. Written informed consent was obtained before data collection.

A total of 98,191 individuals free from ASCVD at baseline were initially included in the current analysis. We further excluded those with missing baseline records on triglyceride (*N* = 66), fasting blood glucose (*N* = 567), body weight (*N* = 35), body height (*N* = 45), or waist circumference (*N* = 2136), reducing the final sample size to 95,342 (Supplementary Fig. 1).

### Data collection and definitions

Participants received face-to-face interviews, laboratory tests and physical examinations during both baseline survey and the following biennial visits. Standardized questionnaires were adopted to collect information on age, gender, sociodemographic characteristics (household income, education attainment and marriage status), health-related life styles (smoking, alcohol intake and physical activity), personal and family medical histories. Blood samples were collected in the morning after an overnight fasting and analyzed with the Hitachi 747 automated analyzer (Tokyo, Japan). Body weight, height, WC and blood pressure were measured by trained medical professionals when participants attended the physical examination.

Current smoking or drinking was defined according to self-reported cigarette or alcohol consumption during the past 12 months. Individuals who exercised more than four times per week (≥ 20 min/time) were classified as active physical activity. Family history of ASCVD was defined as at least one of their parents diagnosed with MI or IS. Hypertension was identified based on systolic/diastolic blood pressure ≥ 140 /90 mmHg, self-reported history of hypertension or antihypertensive medication usage. Diabetes was defined using fasting blood glucose ≥ 7.0 mmol/L, self-reported history of diabetes, treatment with oral hypoglycemic agents or insulin injection. Elevated concentration of total (≥ 6.2 mmol/L) or low-density lipoprotein cholesterol (≥ 4.1 mmol/L), or use of lipid-lowering medication was considered as hyperlipidemia. Chronic Kidney Disease (CKD) was defined as having an estimated glomerular filtration rate (eGFR) < 60 mL/min/1.73 m^2^ and/or having positive proteinuria (≥ 1+) at the baseline survey [24]. 

### Calculation of TyG and its related parameters

The TyG index was calculated as ln (fasting triglyceride [mg/dL] × fasting blood glucose [mg/dL]/2). BMI was calculated as body mass (kg) divided by height squared (m^2^), and WHtR was defined as the ratio of WC (cm) to height (cm). Accordingly, we calculated Tyg-BMI as TyG × BMI, TyG-WC as TyG × WC, and TyG-WHtR as TyG ×WHtR [19]. In addition to baseline measurements, the updated mean of each indicator was further calculated with all available data from baseline to the latest survey before occurrence of ASCVD or to the end of follow-up.

### Assessment of outcomes

Information on disease information and vital status was primarily obtained through face-to-face interviews, biennially updated till the date of death or the latest follow-up visit, whichever came first. Moreover, medical records from the municipal social insurance institution, discharge summaries from local hospitals, and death certificates from provincial vital statistics offices were also checked for additional validation of diagnosis [25]. All causes of events were recorded with the 10th version of the International Classification of Diseases (ICD-10).

The primary outcome for this analysis was the first occurrence of ASCVD, a composite of MI and IS, and both of them were further analyzed separately as secondary outcomes. According to the World Health Organization criteria, MI (ICD-10  *I21–I23*) was identified according to clinical symptom, electrocardiographic manifestation, and concentration of myocardial enzymes; [26] and IS (ICD-10 *I63*) was diagnosed on the basis of clinical signs combined with neuroimages from brain computed tomography or magnetic resonance imaging [27]. 

### Statistical analyses

Baseline characteristics were described as median (inter-quartile range, IQR) for continuous variables and as frequency (%) for categorical variables, stratified by quartiles of baseline TyG index and its related parameters. The potential trends of baseline characteristics across quartiles of TyG and its related parameters were detected using Jonckheere–Terpstra tests. With time-on-study as timescale, the person-years of follow-up was calculated from the completion of baseline survey to either the date of ASCVD, death, or the most recent visit, whichever came first.

The Cox proportional hazards regression model was applied to estimate the hazard ratio (HR) and 95% confidence interval (95% CI) for ASCVD and its subtypes. The Breslow approximation was to handle the resulting ties. The proportional hazard assumption was evaluated by the weighted Schoenfeld residuals [28], and no violations were observed. Models were fitted with adjustments for age, gender, monthly income, education level, marital status, current smoking, current drinking, physical activity, and family history of ASCVD. To explore the potential nonlinear dose-response relationship of TyG index and its related parameters with ASCVD, restricted cubic splines (RCS) were fitted by adjusting for covariates in Model 2, with reference set at the 50th percentile and four knots at the 5th, 35th, 65th, and 95th percentiles of the distribution.

Subgroup analyses by baseline characteristics were conducted for the primary outcome to investigate possible effect modifications. The multiplicative interaction was assessed by introducing a cross-product term, and the *P* value was calculated using the likelihood ratio test. C-index, IDI, and category-free NRI were computed to exploringly quantify and compare the predictive capacity of TyG, TyG-BMI, TyG-WC, and TyG-WHtR, based on the univariable Cox regression models with no other risk factors included. The C-index indicated the probability that a randomly selected participants who experienced ASCVD events during follow-ups had a higher risk score than an individual who had not experienced the event; the IDI quantified the difference in the integrated sensitivity minus that of specificity over all possible cut-off values between two predictive models; and the category-free NRI reflected the improvement in classification rates by one model over the other based upon a continuous risk scale.

To further explore the potential impact of reverse causality, we repeated the primary analysis using a 2-year lag period by excluding ASCVD events that occurred during the initial two years of follow-up. Other sensitivity analyses involved (1) using the updated mean instead of baseline measurements of TyG index and its related parameters; (2) additionally adjusting for hypertension, diabetes and hyperlipidemia; (3) adopting the Cox regression model with time-varying variables updated in each survey before the occurrence of ASCVD events; (4) fitting the Fine & Gary sub-distribution hazard models to further consider the competing risk of non-ASCVD deaths.

The above assessments were performed with SAS version 9.4 (SAS Institute Inc, Cary, NC, USA) and R version 4.1.2 (R Core Team, Vienna, Austria). No adjustments were made for multiple comparison. A two-sided *P* value < 0.05 was considered to be statistically significant.

## Results

### Baseline characteristics of the participants

Of 95,342 eligible participants included, the median age at recruitment was 59.0 years and 75,880 (79.6%) were males. The baseline characteristics stratified by TyG index were shown in Table 1. In comparison with individuals at the lowest quartile of TyG, individuals with higher TyG levels had greater probabilities to be male and less well-educated. Moreover, they were more likely to have a family history of ASCVD and be diagnosed with hypertension, diabetes, hyperlipidemia and CKD. Similar tendency was also observed across quartiles of TyG-BMI, TyG-WC, and TyG-WHtR (Supplementary Tables 1–3).

**Table 1:** Baseline characteristics of participants according to TyG quartiles

	Overall	Quartiles of TyG				
		Quartile 1	Quartile 2	Quartile 3	Quartile 4	*P*_for trend_
Range of TyG		< 8.2	8.2–8.6	8.6–9.1	≥ 9.1	< 0.001
No. of participants	95,342	23,835	23,835	23,832	23,840	< 0.001
Age, years, Median (IQR)	59.0 (51.0, 66.2)	57.9 (49.0, 66.0)	59.0 (51.0, 66.3)	59.2 (52.0, 67.0)	59.0 (52.0, 66.0)	< 0.001
Male, N (%)	75,880 (79.6)	17,556 (73.7)	18,889 (79.2)	19,355 (81.2)	20,080 (84.2)	0.007
High school or above, N (%)	18,674 (19.6)	5642 (23.7)	4469 (18.7)	4430 (18.6)	4133 (17.3)	< 0.001
Monthly income ≥ 600 CNY, N (%)	6151 (6.6)	1676 (7.3)	1461 (6.3)	1499 (6.5)	1515 (6.5)	< 0.001
Married, N (%)	87,453 (94.4)	21,339 (92.6)	22,061 (94.9)	21,956 (94.8)	22,097 (95.4)	< 0.001
Current smoking, N (%)	32,037 (34.3)	7733 (33.4)	7490 (32.0)	8039 (34.4)	8775 (37.6)	0.008
Current drinking, N (%)	35,028 (37.5)	8692 (37.5)	8184 (35.0)	8710 (37.3)	9442 (40.4)	0.008
Active physical activity, N (%)	14,022 (15.2)	3673 (16.0)	3381 (14.6)	3555 (15.4)	3413 (14.8)	< 0.001
Family history of ASCVD, N (%)	10,728 (11.3)	2636 (11.1)	2556 (10.7)	2782 (11.7)	2754 (11.6)	< 0.001
Hypertension, N (%)	32,287 (34.0)	5369 (22.6)	7327 (30.9)	8902 (37.5)	10,689 (45.0)	< 0.001
Diabetes, N (%)	8629 (9.1)	258 (1.1)	606 (2.5)	1774 (7.4)	5991 (25.1)	< 0.001
Hyperlipidemia, N (%)	12,113 (12.7)	1422 (6.0)	2346 (9.8)	3421 (14.4)	4924 (20.7)	< 0.001
CKD, N (%)	18,972 (19.9)	2904 (12.2)	4813 (20.2)	4906 (20.6)	6349 (26.6)	< 0.001
TyG-BMI, Median (IQR)	214.4 (189.0, 242.2)	181.6 (164.8, 200.3)	204.7 (187.4, 223.6)	223.6 (205.1, 243.6)	251.3 (230.2, 274.4)	< 0.001
TyG-WC, Median (IQR)	747.1 (671.6, 830.2)	646.7 (593.1, 703.5)	715.3 (666.2, 767.5)	773.3 (720.8, 830.1)	863.1 (802.6, 929.7)	< 0.001
TyG-WHtR, Median (IQR)	4.5 (4.0, 5.0)	3.9 (3.6, 4.2)	4.3 (4.0, 4.6)	4.6 (4.3, 5.0)	5.1 (4.8, 5.5)	< 0.001

Values were represented by median (IQR) for continuous variables and frequency (percentage) for categorical variables

*ASCVD* atherosclerotic cardiovascular diseases, *BMI* body mass index, *CKD* chronic kidney disease, *IQR* interquartile range, *TyG* triglyceride-glucose, *WC* waist circumference, *WHtR* Waist-to-Height ratio

### Association of TyG and its related parameters with ASCVD

Over a median (IQR) follow-up of 15.0 (14.7, 15.2) years, 8031 (8.4%) cases of ASCVD were identified, including 1,909 (2.0%) MI and 6353 (6.7%) IS. The crude incidence rate of ASCVD increased substantially with the level of TyG and its related parameters. For instance, it ranged from 407.04 to 847.46 per 100,000 person-years across quartiles of TyG and from 328.77 to 945.00 per 100,000 person-years across quartiles of TyG-WHtR. After adjustment for potential confounding factors, higher levels of TyG and its related parameters were still related to elevated risks of ASCVD (Table 2, Supplementary Table 4). More intuitively, RCS curves showed significant positive associations of those indicators with ASCVD and its subtypes, most of which exhibited a nonlinear monotone increase (Supplementary Fig. 2). Per 1 IQR increase in TyG was associated with a 39% higher risk (HR: 1.39; 95% CI 1.35, 1.43) of ASCVD, and the corresponding estimates were even greater for those TyG-related parameters, reaching 1.46 (1.41, 1.50) for TyG-BMI, 1.50 (1.46, 1.55) for TyG-WC, and 1.52 (1.48, 1.57) for TyG-WHtR. In the subtype analyses, similar results were yielded for MI and IS. TyG-WC and TyG-WHtR showed the highest association with MI and IS, respectively. Compared with the lowest quartile, the HR (95% CI) of MI was 1.66 (1.39, 1.97), 2.15 (1.82, 2.54), and 3.05 (2.60, 3.58) for the second, third and fourth quartile of TyG-WC; the HR (95% CI) of IS was 1.35 (1.24, 1.47), 1.64 (1.51, 1.78), and 2.20 (2.04, 2.38) for the second, third and fourth quartile of TyG-WHtR.

**Table 2:** Associations of TyG and its related parameters with atherosclerotic cardiovascular disease and its subtypes

	Quartiles						Continuous
	Quartile 1	Quartile 2	Quartile 3		Quartile 4		Per 1 IQR increase
ASCVD							
TyG							
No of cases (Incidence Rate)^a^	1356 (407.04)	1798 (546.31)	2157 (661.23)		2720 (847.46)		
HR (95% CI)^b^	Reference	1.29 (1.20, 1.39)	1.54 (1.44, 1.65)		2.01 (1.88, 2.15)		1.39 (1.35, 1.43)
TyG-BMI							
No of cases (Incidence Rate)^a^	1292 (390.23)	1822 (553.78)	2186 (670.15)		2731 (845.16)		
HR (95% CI)^b^	Reference	1.34 (1.25, 1.44)	1.60 (1.49, 1.72)		2.12 (1.98, 2.27)		1.46 (1.41, 1.50)
TyG-WC							
No of cases (Incidence Rate)^a^	1086 (322.2)	1783 (539.22)	2194 (674.35)		2968 (938.18)		
HR (95% CI)^b^	Reference	1.44 (1.33, 1.55)	1.69 (1.57, 1.83)		2.30 (2.14, 2.47)		1.50 (1.46, 1.55)
TyG-WHtR							
No of cases (Incidence Rate)^a^	1110 (328.77)	1725 (521.11)	2216 (680.94)		2980 (945.00)		
HR (95% CI)^b^	Reference	1.39 (1.29, 1.51)	1.74 (1.61, 1.87)		2.33 (2.17, 2.50)		1.52 (1.48, 1.57)
Myocardial Infarction							
TyG							
No of cases (Incidence Rate)^a^	276 (81.55)	381 (113.32)	532 (158.89)		720 (217.38)		
HR (95% CI)^b^	Reference	1.35 (1.15, 1.59)	1.85 (1.59, 2.15)		2.56 (2.22, 2.96)		1.51 (1.43, 1.60)
TyG-BMI							
No of cases (Incidence Rate)^a^	284 (84.54)	393 (116.93)	522 (155.85)		710 (212.76)		
HR (95% CI)^b^	Reference	1.30 (1.11, 1.52)	1.67 (1.44, 1.94)		2.42 (2.10, 2.79)		1.55 (1.46, 1.65)
TyG-WC							
No of cases (Incidence Rate)^a^	206 (60.37)	391 (115.75)	534 (160.05)		778 (237.16)		
HR (95% CI)^b^	Reference	1.66 (1.39, 1.97)	2.15 (1.82, 2.54)		3.05 (2.60, 3.58)		1.65 (1.55, 1.75)
TyG-WHtR							
No of cases (Incidence Rate)^a^	224 (65.53)	380 (112.51)	551 (164.94)		754 (230.54)		
HR (95% CI)^b^	Reference	1.52 (1.28, 1.80)	2.10 (1.79, 2.47)		2.83 (2.42, 3.30)		1.64 (1.54, 1.74)
Ischemic Stroke							
TyG							
No of cases (Incidence Rate)^a^	1114 (332.89)	1464 (442.09)	1678 (509.88)		2097 (645.6)		
HR (95% CI)^b^	Reference	1.27 (1.18, 1.38)	1.45 (1.34, 1.57)		1.87 (1.73, 2.01)		1.35 (1.31, 1.40)
TyG-BMI							
No of cases (Incidence Rate)^a^	1038 (312.15)	1473 (444.66)	1724 (523.97)		2118 (647.93)		
HR (95% CI)^b^	Reference	1.35 (1.25, 1.47)	1.58 (1.46, 1.71)		2.03 (1.88, 2.19)		1.43 (1.38, 1.48)
TyG-WC							
No of cases (Incidence Rate)^a^	902 (266.69)	1433 (430.66)	1708 (520.18)		2310 (721)		
HR (95% CI)^b^	Reference	1.38 (1.27, 1.51)	1.58 (1.45, 1.71)		2.14 (1.98, 2.32)		1.46 (1.42, 1.52)
TyG-WHtR							
No of cases (Incidence Rate)^a^	912 (269.12)	1379 (413.98)	1730 (526.89)		2332 (730.17)		
HR (95% CI)^b^	Reference	1.35 (1.24, 1.47)	1.64 (1.51, 1.78)		2.20 (2.04, 2.38)		1.49 (1.44, 1.54)

*ASCVD* atherosclerotic cardiovascular diseases, *BMI* body mass index, *HR (95% CI)* hazard ratio (95% confidence interval), *IQR* interquartile range, *TyG* triglyceride-glucose, *WC* waist circumference, *WHtR* Waist-to-Height ratio

^a^Incidence rate per 100,000 person-years

^b^Cox proportional hazard regression model, adjusted for age, gender, monthly income, education level, marital status, current smoking, current drinking, physical activity, and family history of atherosclerotic cardiovascular diseases

### Subgroup and sensitivity analyses

**Fig. 1:**
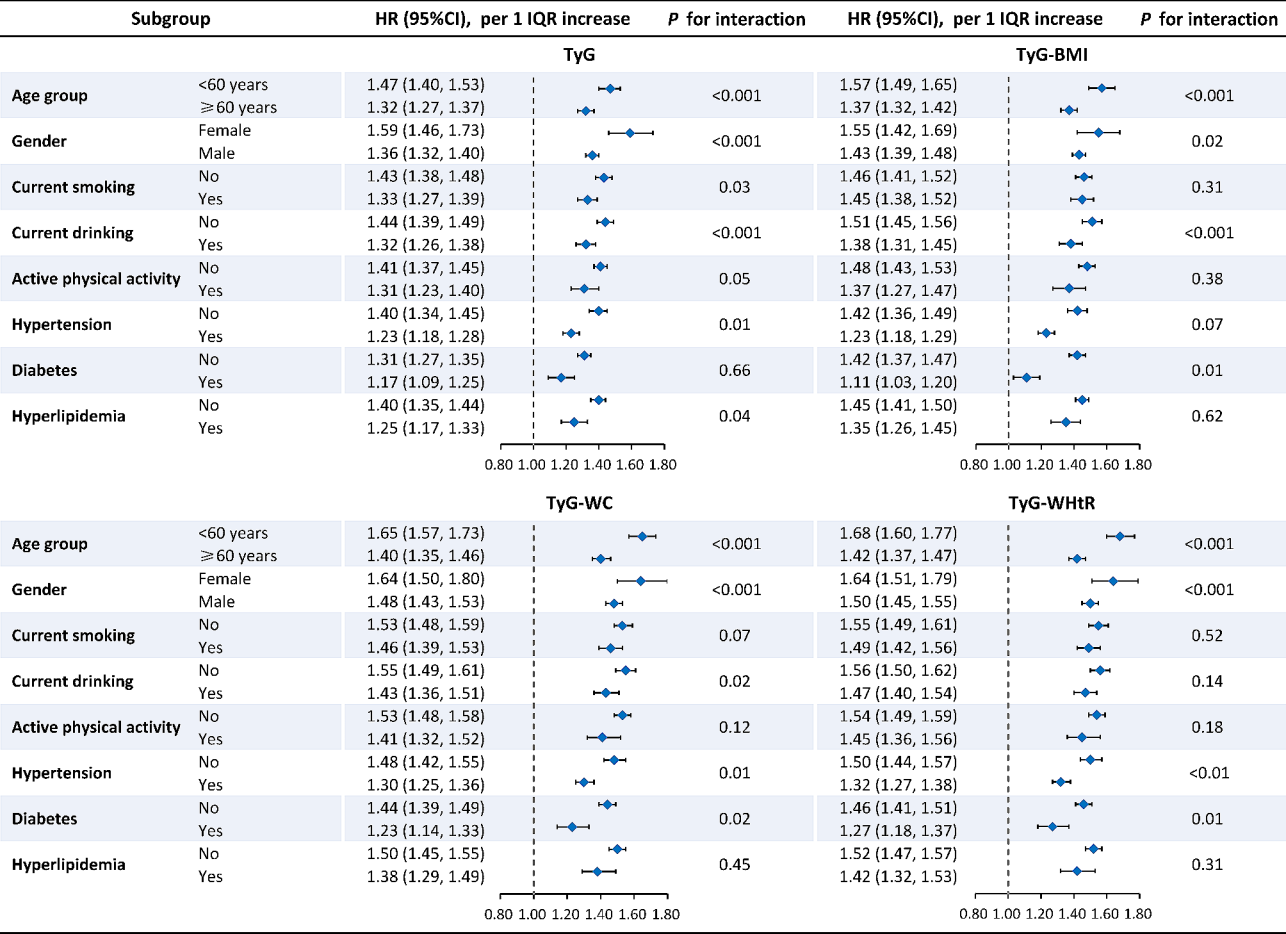
Forest plots of subgroup analyses for the association of TyG and its related parameters with atherosclerotic cardiovascular diseases

The results of subgroup analyses were presented in Fig. 1 and Supplementary Table 5. In general, age and gender significantly interacted with both TyG and its related parameters in relation to ASCVD risk, within which stronger associations were observed among those younger (< 60 years) and female individuals (*P*_for interaction_<0.001). Other significant interactions were also observed between specific subgroups and indicators. For instance, the adverse effect of high TyG-WC and TyG-WHtR on ASCVD seemed to be stronger among those free from hypertension and diabetes.

**Fig. 2:**
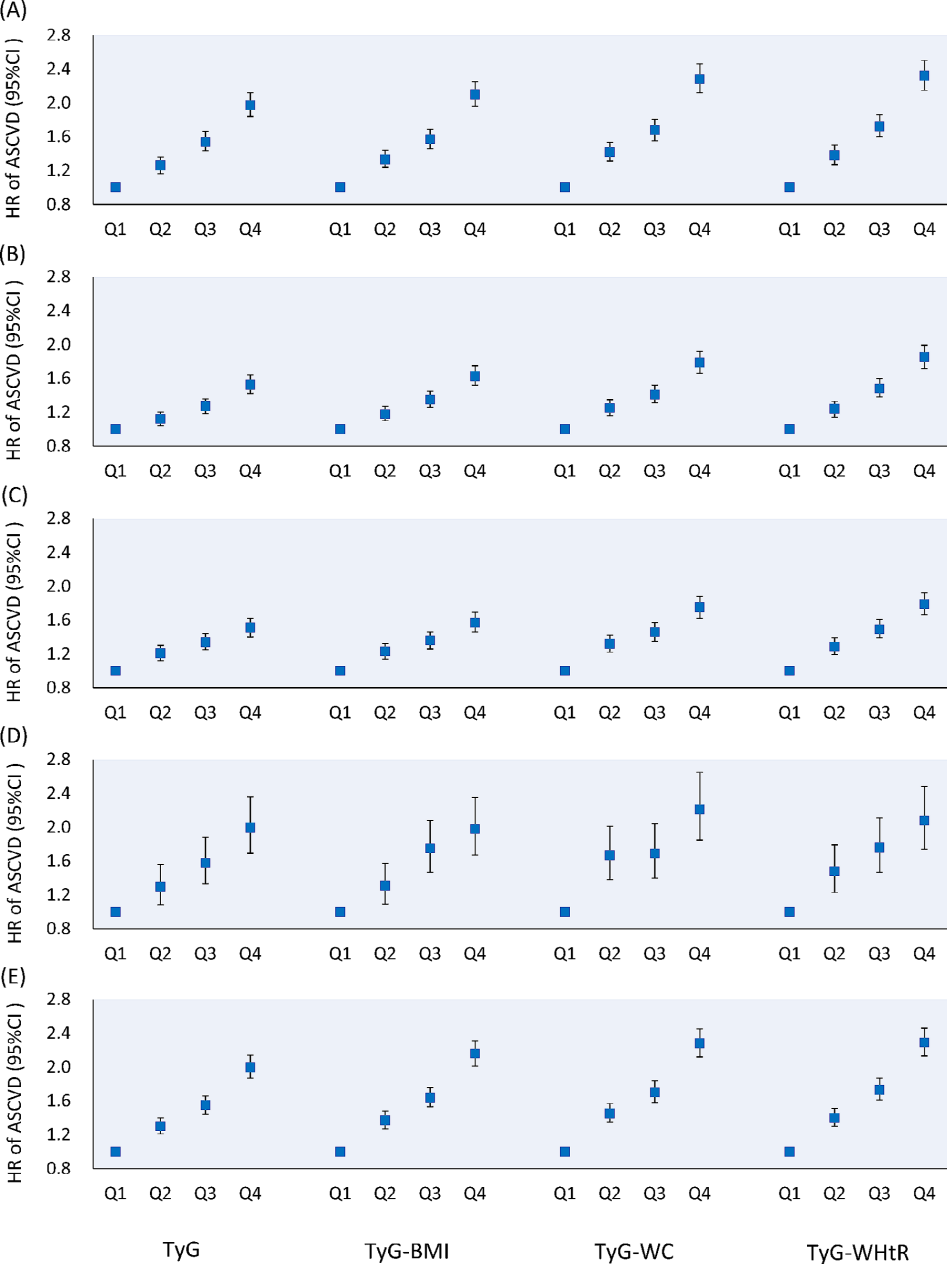
Sensitivity analyses for association of TyG and its related parameters with atherosclerotic cardiovascular diseases. **A** Excluding ASCVD events that occurred during the initial two years of follow-up; **B** Using the updated mean instead of baseline measurements of TyG index and its related parameters; **C** Further adjusting for hypertension, diabetes and hyperlipidemia; **D** Adopting the Cox regression model with time-varying variables updated in each survey before the occurrence of ASCVD events; **E** Adopting the Fine & Gary sub-distribution hazard models to further consider the competing risk of non-ASCVD deaths

Results were consistent when using the updated mean instead of baseline measurements of TyG index and its related parameters; other sensitivity analyses also had no substantial influence on the primary findings (Fig. 2, Supplementary Table 6).

### Predictive values of TyG and its related parameters for ASCVD

The C-index (95% CI) of TyG, TyG-BMI, TyG-WC, and TyG-WHtR was 0.583 (0.577, 0.590), 0.584 (0.578, 0.591), 0.612 (0.606, 0.618), 0.613 (0.607, 0.619) for ASCVD based on the univariable Cox regression models, respectively. In addition, the discriminatory power and risk reclassification of TyG-WC and TyG-WHtR appeared to be slightly better than TyG, with the IDI (95% CI) of 0.004 (0.001, 0.004) and 0.004 (0.001, 0.004), and the category-free NRI (95% CI) of 0.120 (0.025, 0.138) and 0.143 (0.032, 0.166), respectively. However, no significant improvement was observed in the predictive value of TyG-BMI for ASCVD compared with TyG. Similar results were found for MI and IS, and more detailed information was provided in Table 3.

**Table 3:** Reclassification and discrimination statistics for TyG and its related parameters

	C-index (95% CI)	IDI (95% CI)	Category-free NRI (95% CI)
ASCVD			
TyG	0.583 (0.577, 0.590)	Reference	Reference
TyG-BMI	0.584 (0.578, 0.591)	0.000 (– 0.001, 0.000)	0.005 (– 0.024, 0.016)
TyG-WC	0.612 (0.606, 0.618)	0.004 (0.001, 0.004)	0.120 (0.025, 0.138)
TyG-WHtR	0.613 (0.607, 0.619)	0.004 (0.001, 0.004)	0.143 (0.032, 0.166)
Myocardial infarction			
TyG	0.608 (0.595, 0.621)	Reference	Reference
TyG-BMI	0.596 (0.583, 0.609)	0.000 (– 0.001, 0.000)	– 0.038 (– 0.064, 0.017)
TyG-WC	0.635 (0.623, 0.647)	0.001 (0.000, 0.001)	0.122 (0.020, 0.157)
TyG-WHtR	0.631 (0.619, 0.643)	0.001 (0.000, 0.001)	0.113 (0.019, 0.145)
Ischemic stroke			
TyG	0.576 (0.568, 0.583)	Reference	Reference
TyG-BMI	0.581 (0.574, 0.588)	0.000 (0.000, 0.000)	0.020 (0.001, 0.030)
TyG-WC	0.605 (0.598, 0.612)	0.003 (0.000, 0.003)	0.115 (0.025, 0.137)
TyG-WHtR	0.608 (0.601, 0.614)	0.003 (0.001, 0.003)	0.145 (0.034, 0.155)

*ASCVD* atherosclerotic cardiovascular diseases, *BMI* body mass index, *95% CI* 95% confidence interval, *IDI* integrated discrimination improvement, *IQR* interquartile range, *NRI* net reclassification index, *TyG* triglyceride-glucose, *WC* waist circumference, *WHtR* Waist-to-Height ratio

## Discussion

Based on data from the Kailuan cohort, this large-scale, prospective study demonstrated significant positive associations of TyG, TyG-BMI, TyG-WC and TyG-WHtR with the occurrence of ASCVD and its subtypes, which were validated by multiple sensitivity analyses like using the updated mean or time-varying measurements instead of baseline indicators. Although higher TyG and its related parameters were found to be consistently associated with increased risk of ASCVD regardless of age and gender, the effect seemed to be stronger among females and individuals who were < 60 years of age. In addition, TyG-WC and TyG-WHtR have shown better capacity for predicting incident ASCVD compared with single TyG.

The TyG index and its combination with obesity indicators were proposed as simple yet powerful surrogate indicators for IR and metabolic health in clinical applications. Furthermore, emerging epidemiologic evidence has suggested that those indicators were closely related to systematic arterial stiffness, carotid atherosclerosis, coronary artery stenosis, and ASCVD both in the general population and among patients with cardiometabolic diseases like hypertension and diabetes [7, 20, 29–33]. A cross-sectional study conducted in Taiwan, for instance, suggested that individuals with higher TyG, TyG-BMI, and TyG-WC were more likely to have elevated risk of ASCVD [18]. In accordance, two community-based studies in China indicated that the three above-mentioned indicators were positively associated with increased risk of stroke [17, 34]. As for MI, the third National Health and Nutrition Examination Survey (NHANES III) suggested that elevated TyG increased the risk of subclinical myocardial injury [35]. More directly, a previous study based on Kailuan cohort has demonstrated the association between baseline and long-term TyG and occurrence of MI [8]. Nevertheless, it was noteworthy that most of them focused on the influence of the current hottest TyG, while researches on those TyG related parameters remained relatively scattered. Furthermore, few existing studies evaluated those parameters at the same time, thus limiting further comparisons between different indicators. Recently, a cross-sectional study based on an American population has investigated the relationship of TyG and its related indicators with CVD incidence and mortality and evaluated their prediction values comprehensively. However, only a single baseline blood sample was collected in that study, and the diagnosis of CVD was established by self-reported diagnoses without exact time of onset [19]. More importantly, a significant heterogeneity of the association of TyG with CVD between low/middle-income and high-income countries was found in the Prospective Urban Rural Epidemiology (PURE) study, emphasizing the necessity of more evidence from population at diverse levels of economic development [36]. 

To the best of our knowledge, few researches have been conducted in developing countries to compare the correlation between TyG related parameters and ASCVD, of which prospective studies were even rarer. Benefiting from repeated measurements over long-term follow-ups and accurate information on disease incidence, we evaluated and compared the association of TyG, TyG-BMI, TyG-WC, and TyG-WHtR with ASCVD based on a large-scale cohort in China; sensitivity analyses based on long-term average and time-varying levels further confirmed these findings. Consistent with previous publications, we observed significant and positive correlations between all four indicators and ASCVD as well as its subtypes. The HRs of per 1 IQR increase were found to be higher for TyG-WC and TyG-WHtR. Moreover, although the absolute values of C-index estimated based on univariable Cox regression models were relatively lower for the real usefulness in clinical practice, the NRI and IDI indicated a better performance of TyG-WC and TyG-WHtR in predicting risk of ASCVD in comparison with TyG, while no obvious difference was observed between TyG-BMI and TyG. Our findings were generally in line with previous research conducted in high-income countries. For instance, a study from Korea suggested that TyG-WC had a better diagnostic efficacy for coronary artery calcification than TyG-BMI and TyG [37]. Similarly, findings based on the NHANES study also indicated that combining the TyG index with WC and WHtR further enhanced its ability to predict CVD mortality [19]. Mechanisms behind the different predictive performance haven’t been fully explained yet, while the combined effects between IR and excess abdominal fat on the development of ASCVD might be a possible explanation. Both imparted insulin sensitivity, reflecting by TyG, and excessive accumulation of visceral adiposity, indicated by WC and WHtR, were closely related to chronic inflammation, endothelium dysfunction and atherosclerosis, which all contributed to the development of ASCVD. Therefore, novel indicators integrating TyG with central obesity indices might present a more accurate prediction of ASCVD than TyG index alone.

Moreover, our subgroup analyses revealed that the associations of TyG and its related parameters with ASCVD were more pronounced in relatively younger and female individuals. The age-specific influence was consistent with previous studies [19, 38]. One potential reason might be that the elderly were more likely to take multiple medications and be exposed to more cardiovascular risk factors apart from IR or obesity, which to some extent undermined the predictive capability of TyG and other indicators for ASCVD. On the contrary, we found that the interaction of those indices with genders were heterogeneous across studies. For instance, a research conducted in the U.S showed that the relationship between TyG and 10-year risk of ASCVD was stronger in men than women [39]. while the correlation was found to be higher in women than men in another study, in line with our current findings [19]. Therefore, further exploration is essential for deeper explanation of the gender difference.

The advantage of our study merits consideration. The analysis was conducted on the basis of a large-scale, community-based cohort with repeated measurements during 15-year follow-ups, which made it possible to simultaneously compare roles of each indicator in a prospective design, thus helping explore the most suitable risk marker for predicting future risk of ASCVD. Moreover, findings of our study were relatively reliable and robust since no obvious violation was observed with series of sensitivity analyses performed. Nonetheless, our study also has some limitations to address. First, IR indicators including insulin, HbA1c and the Homeostatic Model Assessment for Insulin Resistance (HOMA-IR) were not measured in the Kailuan study, which limited further comparisons with those conventional biomarkers in the current analysis [40]. Secondly, in spite of adequate confounding adjustments in the analyses, residual or unmeasured confounding could not be completely avoided given the observational study design. Third, since only information on MI and IS was collected during follow-ups, the incidence rate of ASCVD might be underestimated in the current analyses, and we were unable to explore the association of TyG and its related parameters with other ASCVDs. Moreover, generalizing of our findings to other populations with diverse time and social background should be cautious since only individuals living in Northern China were included in the current study.

## Conclusion

The TyG index and TyG related parameters, namely TyG-BMI, TyG-WC and TyG-WHtR, were significantly associated with the incidence of ASCVD and its major subtypes MI and IS. In addition, we demonstrated that innovative indices combining the TyG index with central obesity parameters WC, and WHtR outperformed TyG alone in prediction for incidence of ASCVD. In consideration of the increasingly severe disease burden and limited medical resource, our findings of convenient, low-cost surrogate indices of IR with good predictive capacity are expected to provide some valuable references for future ASCVD prevention in low- and middle-income counties including China.

### Electronic supplementary material

Below is the link to the electronic supplementary material.

Supplementary Table 1. Baseline characteristics of participants according to TyG-BMI quartiles. Supplementary Table 2. Baseline characteristics of participants according to TyG-WC quartiles. Supplementary Table 3. Baseline characteristics of participants according to TyG-WHtR quartiles. Supplementary Table 4. Coefficient (95%CI) of each covariate included in the Cox regression models. Supplementary Table 5. Subgroup analyses of TyG and its related parameters with atherosclerotic cardiovascular disease. Supplementary Table 6. Sensitivity analyses for association of TyG and its related parameters with atherosclerotic cardiovascular disease and its subtypes. Supplementary Figure 1. Flow chart of participants included and excluded in the analyses. Supplementary Figure 2. Restricted cubic spline curves of the association of triglyceride-glucose and its related parameters with atherosclerotic cardiovascular disease and its subtypes.Supplementary Material 1

## Data Availability

The datasets used and/or analyzed during the current study are available from the corresponding author on reasonable request.

## Abbreviations

ASCVD: Atherosclerotic cardiovascular diseases
BMI: Body mass index
CI: Confidence interval
HR: Hazard ratio
IDI: Integrated discrimination improvement
IQR: Interquartile range
IR: Insulin resistance
IS: Ischemic stroke
MI: Myocardial infarction
NRI: Net reclassification index
RCS: Restricted cubic splines
TyG: Triglyceride-glucose
WC: Waist circumference
WHtR: Waist-to-Height ratio
