# Supplementary material for: Association of triglyceride-glucose index and its related parameters with atherosclerotic cardiovascular disease: evidence from a 15-year follow-up of Kailuan cohort
Source: Cardiovasc Diabetol. 2024 Jun 19;23:208. doi: 10.1186/s12933-024-02290-3 (PMC11188278; doi:10.1186/s12933-024-02290-3)
Supplement: Supplementary file 1 — Supplementary Table 1. Baseline characteristics of participants according to TyG-BMI quartiles. Supplementary Table 2. Baseline characteristics of participants according to TyG-WC quartiles. Supplementary Table 3. Baseline characteristics of participants according to TyG-WHtR quartiles. Supplementary Table 4. Coefficient (95%CI) of each covariate included in the Cox regression models. Supplementary Table 5. Subgroup analyses of TyG and its related parameters with atherosclerotic cardiovascular disease. Supplementary Table 6. Sensitivity analyses for association of TyG and its related parameters with atherosclerotic cardiovascular disease and its subtypes. Supplementary Figure 1. Flow chart of participants included and excluded in the analyses. Supplementary Figure 2. Restricted cubic spline curves of the association of triglyceride-glucose and its related parameters with atherosclerotic cardiovascular disease and its subtypes.Supplementary Material 1 [file 12933_2024_2290_MOESM1_ESM.pdf]

Association of triglyceride-glucose index and its related parameters with atherosclerotic cardiovascular disease: evidence from a 15-year follow-up of Kailuan cohort

Additional files

Supplementary Table 1. Baseline characteristics of participants according to TyG-BMI quartiles. 2
Supplementary Table 2. Baseline characteristics of participants according to TyG-WC quartiles. 4
Supplementary Table 3. Baseline characteristics of participants according to TyG-WHtR quartiles. 6
Supplementary Table 4. Coefficient (95%CI) of each covariate included in the Cox regression models 8
Supplementary Table 5. Subgroup analyses of TyG and its related parameters with atherosclerotic cardiovascular disease. 14
Supplementary Table 6. Sensitivity analyses for association of TyG and its related parameters with atherosclerotic cardiovascular disease and its subtypes. 20
Supplementary Figure 1. Flow chart of participants included and excluded in the analyses. 26
Supplementary Figure 2. Restricted cubic spline curves of the association of triglyceride-glucose and its related parameters with atherosclerotic cardiovascular disease and its subtypes. 27

Supplementary Table 1. Baseline characteristics of participants according to TyG-BMI quartiles.

	Overall	Quartiles of TyG-BMI				
		Quartile 1	Quartile 2	Quartile 3	Quartile 4	*P* for trend
Range of TyG-BMI		<189.0	189.0-214.4	214.4-242.2	≥242.2	
No. of participants	95342	23835	23836	23835	23836	
Age, years, Median (IQR)	59.0 (51.0, 66.2)	57.2 (48.7, 66.0)	59.0 (51.4, 66.1)	59.7 (52.0, 67.0)	59.0 (51.1, 66.0)	<0.001
Male, N (%)	75880 (79.6)	17397 (73.0)	19078 (80.0)	19719 (82.7)	19686 (82.6)	<0.001
High school or above, N (%)	18674 (19.6)	5701 (23.9)	4416 (18.5)	4304 (18.1)	4253 (17.8)	<0.001
Monthly income ≥ 600 CNY, N (%)	6151 (6.6)	1583 (6.8)	1502 (6.5)	1579 (6.8)	1487 (6.4)	0.210
Married, N (%)	87453 (94.4)	21470 (92.6)	21886 (94.6)	22002 (95.2)	22095 (95.3)	<0.001
Current smoking, N (%)	32037 (34.3)	7964 (34.2)	7982 (34.3)	8010 (34.4)	8081 (34.6)	0.396
Current drinking, N (%)	35028 (37.5)	8443 (36.2)	8727 (37.5)	8826 (37.8)	9032 (38.6)	<0.001
Active physical activity, N (%)	14022 (15.2)	3387 (14.6)	3551 (15.4)	3537 (15.3)	3547 (15.3)	0.057

	Overall	Quartiles of TyG-BMI				
		Quartile 1	Quartile 2	Quartile 3	Quartile 4	*P* for trend
Family history of ASCVD, N (%)	10728 (11.3)	2458 (10.3)	2667 (11.2)	2755 (11.6)	2848 (11.9)	<0.001
Hypertension, N (%)	32287 (34.0)	4418 (18.6)	6993 (29.5)	9147 (38.5)	11729 (49.3)	<0.001
Diabetes, N (%)	8629 (9.1)	548 (2.3)	1216 (5.1)	2263 (9.5)	4602 (19.3)	<0.001
Hyperlipidemia, N (%)	12113 (12.7)	1787 (7.5)	2618 (11.0)	3366 (14.1)	4342 (18.2)	<0.001
CKD, N (%)	18972 (19.9)	3307 (13.9)	4512 (18.9)	5126 (21.5)	6027 (25.3)	<0.001
TyG, Median (IQR)	8.6 (8.2, 9.1)	8.1 (7.8, 8.4)	8.4 (8.2, 8.7)	8.7 (8.4, 9.1)	9.2 (8.8, 9.7)	<0.001
TyG-WC, Median (IQR)	747.1 (671.6, 830.2)	632.6 (585.8, 682.8)	713.6 (673.9, 756.2)	779.0 (735.7, 824.3)	872.4 (816.8, 933.2)	<0.001
TyG-WHtR, Median (IQR)	4.5 (4.0, 5.0)	3.8 (3.5, 4.1)	4.3 (4.0, 4.5)	4.6 (4.4, 4.9)	5.2 (4.9, 5.6)	<0.001

Values were represented by median (IQR) for continuous variables and frequency (percentage) for categorical variables.

Abbreviations: ASCVD, atherosclerotic cardiovascular disease; BMI, body mass index; CKD, chronic kidney disease; IQR, interquartile range; TyG, triglyceride-glucose; WC, waist circumference; WHtR, Waist-to-Height ratio.

Supplementary Table 2. Baseline characteristics of participants according to TyG-WC quartiles.

	Overall	Quartiles of TyG-WC				
		Quartile 1	Quartile 2	Quartile 3	Quartile 4	*P* for trend
Range of TyG-WC		<671.6	671.6-747.1	747.1-830.2	≥830.2	
No. of participants	95342	23835	23836	23835	23836	
Age, years, Median (IQR)	59.0 (51.0, 66.2)	55.0 (47.0, 64.0)	58.8 (51.0, 66.0)	60.0 (52.5, 67.0)	60.7 (53.0, 68.0)	<0.001
Male, N (%)	75880 (79.6)	15901 (66.7)	19179 (80.5)	20133 (84.5)	20667 (86.7)	<0.001
High school or above, N (%)	18674 (19.6)	6705 (28.1)	4120 (17.3)	4055 (17.0)	3794 (15.9)	<0.001
Monthly income ≥ 600 CNY, N (%)	6151 (6.6)	1745 (7.4)	1404 (6.0)	1528 (6.6)	1474 (6.5)	0.001
Married, N (%)	87453 (94.4)	21648 (92.3)	22082 (95.0)	21967 (95.1)	21756 (95.3)	<0.001
Current smoking, N (%)	32037 (34.3)	7588 (32.3)	7887 (33.8)	8256 (35.5)	8306 (35.9)	<0.001
Current drinking, N (%)	35028 (37.5)	8387 (35.7)	8514 (36.4)	8995 (38.6)	9132 (39.4)	<0.001
Active physical activity, N (%)	14022 (15.2)	3509 (15.0)	3499 (15.1)	3644 (15.8)	3370 (14.8)	0.820

	Overall	Quartiles of TyG-WC				
		Quartile 1	Quartile 2	Quartile 3	Quartile 4	*P* for trend
Family history of ASCVD, N (%)	10728 (11.3)	2833 (11.9)	2608 (10.9)	2655 (11.1)	2632 (11.0)	0.011
Hypertension, N (%)	32287 (34.0)	4322 (18.2)	7306 (30.8)	9174 (38.6)	11485 (48.4)	<0.001
Diabetes, N (%)	8629 (9.1)	424 (1.8)	1110 (4.7)	2147 (9.0)	4948 (20.8)	<0.001
Hyperlipidemia, N (%)	12113 (12.7)	1712 (7.2)	2643 (11.1)	3314 (13.9)	4444 (18.6)	<0.001
CKD, N(%)	18972 (19.9)	3416 (14.3)	4664 (19.6)	5021 (21.1)	5871 (24.6)	<0.001
TyG, Median (IQR)	8.6 (8.2, 9.1)	8.1 (7.8, 8.3)	8.4 (8.2, 8.7)	8.7 (8.4, 9.1)	9.3 (8.9, 9.7)	<0.001
TyG-BMI, Median (IQR)	214.4 (189.0, 242.2)	177.8 (163.4, 192.1)	204.6 (191.3, 218.7)	226.1 (211.1, 241.2)	256.6 (237.2, 277.8)	<0.001
TyG-WHtR, Median (IQR)	4.5 (4.0, 5.0)	3.7 (3.5, 3.9)	4.2 (4.1, 4.4)	4.7 (4.5, 4.8)	5.3 (5.1, 5.6)	<0.001

Values were represented by median (IQR) for continuous variables and frequency (percentage) for categorical variables.

Abbreviations: ASCVD, atherosclerotic cardiovascular disease; BMI, body mass index; CKD, chronic kidney disease; IQR, interquartile range; TyG, triglyceride-glucose; WC, waist circumference; WHtR, Waist-to-Height ratio.

Supplementary Table 3. Baseline characteristics of participants according to TyG-WHtR quartiles.

	Overall	Quartiles of TyG-WHtR				
		Quartile 1	Quartile 2	Quartile 3	Quartile 4	***P* for trend**
Range of TyG- WHtR		<4.0	4.0-4.5	4.5-5.0	≥5.0	
No. of participants	95342	23835	23836	23835	23836	
Age, years, Median (IQR)	59.0 (51.0, 66.2)	55.0 (46.0, 63.0)	58.0 (51.0, 65.8)	60.0 (52.5, 67.0)	61.5 (54.0, 68.8)	<0.001
Male, N (%)	75880 (79.6)	17644 (74.0)	19494 (81.8)	19738 (82.8)	19004 (79.7)	<0.001
High school or above, N (%)	18674 (19.6)	6715 (28.2)	4260 (17.9)	4074 (17.1)	3625 (15.2)	<0.001
Monthly income ≥ 600 CNY, N (%)	6151 (6.6)	1748 (7.5)	1425 (6.1)	1489 (6.5)	1489 (6.5)	<0.001
Married, N (%)	87453 (94.4)	21661 (92.4)	22142 (95.2)	21990 (95.3)	21660 (94.9)	<0.001
Current smoking, N (%)	32037 (34.3)	8309 (35.4)	7951 (34.0)	8210 (35.3)	7567 (32.7)	<0.001
Current drinking, N (%)	35028 (37.5)	9138 (38.9)	8608 (36.8)	8974 (38.5)	8308 (35.9)	<0.001
Active physical activity, N (%)	14022 (15.2)	3485 (14.9)	3343 (14.4)	3599 (15.6)	3595 (15.8)	<0.001

	Overall	Quartiles of TyG-WHtR				
		**Quartile 1**	**Quartile 2**	**Quartile 3**	**Quartile 4**	***P* for trend**
Family history of ASCVD, N (%)	10728 (11.3)	2750 (11.5)	2560 (10.7)	2800 (11.7)	2618 (11.0)	0.474
Hypertension, N (%)	32287 (34.0)	4320 (18.2)	7294 (30.7)	9086 (38.3)	11587 (48.8)	<0.001
Diabetes, N (%)	8629 (9.1)	408 (1.7)	1045 (4.4)	2179 (9.1)	4997 (21.0)	<0.001
Hyperlipidemia, N (%)	12113 (12.7)	1688 (7.1)	2524 (10.6)	3360 (14.1)	4541 (19.1)	<0.001
CKD, N(%)	18972 (19.9)	3312 (13.9)	4683 (19.7)	4861 (20.4)	6116 (25.7)	<0.001
TyG, Median (IQR)	8.6 (8.2, 9.1)	8.1 (7.8, 8.3)	8.4 (8.2, 8.7)	8.8 (8.4, 9.1)	9.3 (8.9, 9.7)	<0.001
TyG-BMI, Median (IQR)	214.4 (189.0, 242.2)	177.8 (163.4, 191.8)	205.1 (191.7, 218.9)	226.4 (211.2, 241.4)	256.9 (237.3, 278.0)	<0.001
TyG-WC, Median (IQR)	747.1 (671.6, 830.2)	620.8 (582.0, 654.0)	712.3 (688.2, 737.1)	786.0 (758.8, 813.9)	890.8 (851.6, 942.1)	<0.001

Values were represented by median (IQR) for continuous variables and frequency (percentage) for categorical variables.

Abbreviations: ASCVD, atherosclerotic cardiovascular disease; BMI, body mass index; CKD, chronic kidney disease; IQR, interquartile range; TyG, triglyceride-glucose; WC, waist circumference; WHtR, Waist-to-Height ratio.

Supplementary Table 4. Coefficient (95%CI) of each covariate included in the Cox regression models. [a]

	Reference	Category	ASCVD		MI		CI	
			Coefficient(95%CI)	*P*	Coefficient(95%CI)	*P*	Coefficient(95%CI)	*P*
**TyG**								
TyG	Quartile 1	Quartile 2	0.26(0.18,0.33)	<0.001	0.30(0.14,0.46)	<0.001	0.24(0.16,0.32)	<0.001
TyG	Quartile 1	Quartile 3	0.43(0.36,0.50)	<0.001	0.62(0.47,0.77)	<0.001	0.37(0.29,0.45)	<0.001
TyG	Quartile 1	Quartile 4	0.70(0.63,0.76)	<0.001	0.94(0.80,1.09)	<0.001	0.63(0.55,0.70)	<0.001
Age			0.05(0.04,0.05)	<0.001	0.05(0.04,0.05)	<0.001	0.04(0.04,0.05)	<0.001
Gender	Female	Male	0.65(0.57,0.72)	<0.001	0.84(0.67,1.00)	<0.001	0.61(0.52,0.69)	<0.001
Education level	Illiteracy to Middle school	High school or above	-0.34(-0.42, -0.27)	<0.001	-0.22(-0.36, -0.07)	0.004	-0.39(-0.47, -0.31)	<0.001
Monthly income	<600 CNY	⩾ 600 CNY	-0.11(-0.21, -0.01)	0.04	-0.06(-0.26,0.15)	0.60	-0.13(-0.25, -0.02)	0.02
Marital status	Other situations	Married	0.03(-0.08,0.13)	0.65	0.07(-0.15,0.30)	0.51	0.01(-0.11,0.13)	0.82
Current smoking	No	Yes	0.22(0.17,0.28)	<0.001	0.35(0.24,0.46)	<0.001	0.19(0.12,0.25)	<0.001

	Reference	Category	ASCVD		MI		CI	
			**Coefficient(95%CI)**	***P***	**Coefficient(95%CI)**	***P***	**Coefficient(95%CI)**	***P***
Current drinking	No	Yes	-0.16(-0.22, -0.10)	<0.001	-0.42(-0.54, -0.31)	0.000	-0.09(-0.15, -0.03)	0.005
Physical activity	Inactive	Active	0.02(-0.04,0.08)	0.46	0.01(-0.11,0.13)	0.87	0.02(-0.05,0.09)	0.56
Family history of ASCVD	No	Yes	0.08(0.01,0.16)	0.02	0.02(-0.13,0.18)	0.77	0.10(0.02,0.18)	0.02
**TyG-BMI**								
TyG-BMI	Quartile 1	Quartile 2	0.29(0.22,0.37)	<0.001	0.26(0.10,0.42)	0.001	0.30(0.22,0.38)	<0.001
TyG-BMI	Quartile 1	Quartile 3	0.47(0.40,0.54)	<0.001	0.51(0.36,0.66)	<0.001	0.46(0.38,0.54)	<0.001
TyG-BMI	Quartile 1	Quartile 4	0.75(0.68,0.82)	<0.001	0.89(0.74,1.03)	<0.001	0.71(0.63,0.79)	<0.001
Age			0.05(0.04,0.05)	<0.001	0.05(0.04,0.05)	<0.001	0.04(0.04,0.05)	<0.001
Gender	Female	Male	0.64(0.57,0.72)	<0.001	0.83(0.67,1.00)	<0.001	0.60(0.51,0.68)	<0.001
Education level	Illiteracy to Middle school	High school or above	-0.34(-0.41, -0.27)	<0.001	-0.21(-0.36, -0.07)	0.004	-0.39(-0.47, -0.30)	<0.001

	Reference	Category	ASCVD		MI		CI	
			Coefficient(95%CI)	*P*	Coefficient(95%CI)	*P*	Coefficient(95%CI)	*P*
Monthly income	<600 CNY	⩾ 600 CNY	-0.12(-0.22, -0.01)	0.03	-0.06(-0.27,0.14)	0.55	-0.14(-0.26, -0.03)	0.02
Marital status	Other situations	Married	0.03(-0.08,0.13)	0.65	0.08(-0.15,0.30)	0.50	0.01(-0.11,0.13)	0.84
Current smoking	No	Yes	0.25(0.19,0.31)	<0.001	0.37(0.26,0.49)	0.000	0.21(0.15,0.27)	<0.001
Current drinking	No	Yes	-0.16(-0.22, -0.11)	<0.001	-0.42(-0.54, -0.31)	0.000	-0.09(-0.16, -0.03)	0.003
Physical activity	Inactive	Active	0.01(-0.05,0.07)	0.76	-0.01(-0.13,0.12)	0.92	0.01(-0.06,0.08)	0.81
Family history of ASCVD	No	Yes	0.07(0.00,0.15)	0.05	0.01(-0.15,0.17)	0.90	0.09(0.01,0.17)	0.03
**TyG-WC**								
TyG-WC	Quartile 1	Quartile 2	0.36(0.29,0.44)	<0.001	0.50(0.33,0.68)	0.000	0.33(0.24,0.41)	<0.001

	Reference	Category	ASCVD		MI		CI	
			**Coefficient(95%CI)**	***P***	**Coefficient(95%CI)**	***P***	**Coefficient(95%CI)**	***P***
TyG-WC	Quartile 1	Quartile 3	0.53(0.45,0.60)	<0.001	0.77(0.60,0.93)	0.000	0.46(0.37,0.54)	<0.001
TyG-WC	Quartile 1	Quartile 4	0.83(0.76,0.90)	<0.001	1.12(0.96,1.28)	0.000	0.76(0.68,0.84)	<0.001
Age			0.04(0.04,0.04)	<0.001	0.04(0.04,0.05)	0.000	0.04(0.04,0.04)	<0.001
Gender	Female	Male	0.58(0.50,0.66)	<0.001	0.75(0.58,0.91)	0.000	0.54(0.46,0.63)	<0.001
Education level	Illiteracy to Middle school	High school or above	-0.33(-0.40, -0.26)	<0.001	-0.20(-0.35, -0.06)	0.007	-0.38(-0.46, -0.30)	<0.001
Monthly income	<600 CNY	⩾ 600 CNY	-0.12(-0.22, -0.02)	0.02	-0.07(-0.28,0.14)	0.52	-0.14(-0.26, -0.03)	0.02
Marital status	Other situations	Married	0.02(-0.08,0.13)	0.65	0.08(-0.15,0.30)	0.51	0.01(-0.11,0.13)	0.83
Current smoking	No	Yes	0.24(0.19,0.30)	<0.001	0.37(0.26,0.49)	0.000	0.20(0.14,0.27)	<0.001
Current drinking	No	Yes	-0.17(-0.22, -0.11)	<0.001	-0.43(-0.54, -0.31)	0.000	-0.09(-0.16, -0.03)	0.003

	Reference	Category	ASCVD		MI		CI	
			**Coefficient(95%CI)**	***P***	**Coefficient(95%CI)**	***P***	**Coefficient(95%CI)**	***P***
Physical activity	Inactive	Active	0.04(-0.02,0.10)	0.23	0.03(-0.09,0.15)	0.63	0.03(-0.03,0.10)	0.33
Family history of ASCVD	No	Yes	0.08(0.01,0.16)	0.03	0.02(-0.14,0.17)	0.81	0.10(0.02,0.18)	0.02
**TyG-WHtR**								
TyG-WHtR	Quartile 1	Quartile 2	0.33(0.26,0.41)	<0.001	0.42(0.25,0.59)	0.000	0.30(0.22,0.39)	<0.001
TyG-WHtR	Quartile 1	Quartile 3	0.55(0.48,0.63)	<0.001	0.74(0.58,0.90)	0.000	0.50(0.41,0.58)	<0.001
TyG-WHtR	Quartile 1	Quartile 4	0.85(0.78,0.92)	<0.001	1.04(0.89,1.20)	0.000	0.79(0.71,0.87)	<0.001
Age			0.04(0.04,0.04)	<0.001	0.04(0.04,0.05)	0.000	0.04(0.04,0.04)	<0.001
Gender	Female	Male	0.68(0.61,0.76)	<0.001	0.88(0.71,1.04)	0.000	0.64(0.56,0.72)	<0.001
Education level	Illiteracy to Middle school	High school or above	-0.32(-0.40, -0.25)	<0.001	-0.19(-0.34, -0.05)	0.01	-0.37(-0.45, -0.29)	<0.001
Monthly income	<600 CNY	⩾ 600 CNY	-0.12(-0.22, -0.02)	0.02	-0.07(-0.27,0.14)	0.53	-0.14(-0.26, -0.03)	0.02

	Reference	Category	ASCVD		MI		CI	
			**Coefficient(95%CI)**	***P***	**Coefficient(95%CI)**	***P***	**Coefficient(95%CI)**	***P***
Marital status	Other situations	Married	0.04(-0.07,0.14)	0.52	0.09(-0.13,0.31)	0.43	0.02(-0.10,0.14)	0.71
Current smoking	No	Yes	0.24(0.19,0.30)	<0.001	0.37(0.25,0.48)	0.000	0.20(0.14,0.26)	<0.001
Current drinking	No	Yes	-0.16(-0.22, -0.11)	<0.001	-0.42(-0.54, -0.31)	0.000	-0.09(-0.16, -0.03)	0.004
Physical activity	Inactive	Active	0.03(-0.03,0.09)	0.27	0.02(-0.10,0.15)	0.70	0.03(-0.04,0.10)	0.36
Family history of ASCVD	No	Yes	0.08(0.01,0.16)	0.02	0.02(-0.13,0.18)	0.79	0.10(0.02,0.18)	0.02

[a] Cox proportional hazard regression model, adjusted for age, gender, monthly income, education level, marital status, current smoking, current drinking, physical activity, and family history of atherosclerotic cardiovascular diseases.

Abbreviations: ASCVD, atherosclerotic cardiovascular diseases; BMI, body mass index; HR (95%CI), hazard ratio (95% confidence interval); IQR, interquartile range; TyG, triglyceride-glucose; WC, waist circumference; WHtR, Waist-to-Height ratio.

Supplementary Table 5. Subgroup analyses of TyG and its related parameters with atherosclerotic cardiovascular disease.

HR (95%CI) [a]		Quartiles				Continuous	P for interaction
		Quartile 1	Quartile 2	Quartile 3	Quartile 4	Per 1 IQR Increase	
**TyG**							
Age group	<60 years	Reference	1.54(1.35,1.75)	1.77(1.56,2.01)	2.48(2.20,2.79)	1.47(1.40,1.53)	<0.001
	≥60 years	Reference	1.16(1.07,1.27)	1.40(1.29,1.52)	1.72(1.59,1.87)	1.32(1.27,1.37)	
Gender	Female	Reference	1.37(1.08,1.73)	1.86(1.49,2.32)	2.72(2.19,3.37)	1.59(1.46,1.73)	<0.001
	Male	Reference	1.27(1.18,1.37)	1.49(1.38,1.60)	1.90(1.77,2.04)	1.36(1.32,1.40)	
Current smoking	No	Reference	1.28(1.16,1.40)	1.57(1.43,1.72)	2.12(1.94,2.31)	1.43(1.38,1.48)	0.03
	Yes	Reference	1.31(1.17,1.47)	1.49(1.34,1.67)	1.82(1.64,2.03)	1.33(1.27,1.39)	
Current drinking	No	Reference	1.33(1.21,1.46)	1.66(1.51,1.81)	2.20(2.02,2.40)	1.44(1.39,1.49)	<0.001
	Yes	Reference	1.25(1.11,1.40)	1.38(1.23,1.54)	1.74(1.56,1.93)	1.32(1.26,1.38)	
Active physical activity	No	Reference	1.35(1.24,1.46)	1.58(1.46,1.71)	2.09(1.94,2.26)	1.41(1.37,1.45)	0.05
	Yes	Reference	1.09(0.93,1.28)	1.42(1.22,1.65)	1.69(1.46,1.96)	1.31(1.23,1.40)	
Hypertension	No	Reference	1.25(1.13,1.38)	1.49(1.35,1.64)	1.98(1.80,2.18)	1.40(1.34,1.45)	0.01
	Yes	Reference	1.17(1.06,1.31)	1.29(1.17,1.43)	1.53(1.39,1.68)	1.23(1.18,1.28)	

HR (95%CI) [a]		Quartiles				Continuous	P for interaction
		Quartile 1	Quartile 2	Quartile 3	Quartile 4	Per 1 IQR Increase	
Diabetes	No	Reference	1.27(1.18,1.37)	1.49(1.39,1.60)	1.73(1.60,1.86)	1.31(1.27,1.35)	0.66
	Yes	Reference	1.38(0.90,2.13)	1.35(0.91,2.02)	1.66(1.13,2.43)	1.17(1.09,1.25)	
Hyperlipidemia	No	Reference	1.31(1.21,1.42)	1.54(1.43,1.66)	2.01(1.87,2.16)	1.40(1.35,1.44)	0.04
	Yes	Reference	0.99(0.80,1.23)	1.20(0.98,1.46)	1.46(1.21,1.77)	1.25(1.17,1.33)	
**TyG-BMI**							
Age group	<60 years	Reference	1.62(1.42,1.84)	1.92(1.69,2.18)	2.61(2.31,2.94)	1.57(1.49,1.65)	<0.001
	≥60 years	Reference	1.19(1.09,1.30)	1.40(1.29,1.53)	1.83(1.68,1.99)	1.37(1.32,1.42)	
Gender	Female	Reference	1.33(1.06,1.68)	1.80(1.45,2.24)	2.50(2.03,3.08)	1.55(1.42,1.69)	0.02
	Male	Reference	1.33(1.23,1.44)	1.56(1.44,1.68)	2.04(1.90,2.19)	1.43(1.39,1.48)	
Current smoking	No	Reference	1.37(1.25,1.51)	1.69(1.54,1.85)	2.20(2.01,2.40)	1.46(1.41,1.52)	0.31
	Yes	Reference	1.30(1.16,1.46)	1.48(1.32,1.65)	2.00(1.80,2.23)	1.45(1.38,1.52)	
Current drinking	No	Reference	1.42(1.29,1.56)	1.76(1.61,1.93)	2.32(2.13,2.54)	1.51(1.45,1.56)	<0.001
	Yes	Reference	1.24(1.10,1.39)	1.38(1.23,1.54)	1.84(1.65,2.05)	1.38(1.31,1.45)	
	No	Reference	1.37(1.26,1.48)	1.63(1.50,1.76)	2.18(2.02,2.35)	1.48(1.43,1.53)	0.38

HR (95%CI) [a]		Quartiles				Continuous	P for interaction
		Quartile 1	Quartile 2	Quartile 3	Quartile 4	Per 1 IQR Increase	
Active physical activity	Yes	Reference	1.25(1.06,1.47)	1.49(1.27,1.75)	1.86(1.60,2.17)	1.37(1.27,1.47)	
Hypertension	No	Reference	1.27(1.15,1.40)	1.51(1.37,1.66)	1.94(1.76,2.14)	1.42(1.36,1.49)	0.07
	Yes	Reference	1.19(1.06,1.33)	1.28(1.15,1.43)	1.54(1.38,1.71)	1.23(1.18,1.29)	
Diabetes	No	Reference	1.30(1.21,1.41)	1.53(1.42,1.64)	1.97(1.83,2.12)	1.42(1.37,1.47)	0.01
	Yes	Reference	1.31(0.98,1.75)	1.32(1.00,1.74)	1.41(1.09,1.84)	1.11(1.03,1.20)	
Hyperlipidemia	No	Reference	1.35(1.25,1.46)	1.60(1.48,1.73)	2.10(1.95,2.26)	1.45(1.41,1.50)	0.62
	Yes	Reference	1.19(0.97,1.47)	1.39(1.15,1.69)	1.80(1.50,2.17)	1.35(1.26,1.45)	
**TyG-WC**							
Age group	<60 years	Reference	1.63(1.43,1.86)	2.07(1.82,2.36)	2.91(2.57,3.29)	1.65(1.57,1.73)	<0.001
	≥60 years	Reference	1.29(1.17,1.42)	1.45(1.32,1.59)	1.92(1.76,2.10)	1.40(1.35,1.46)	
Gender	Female	Reference	1.79(1.44,2.23)	2.49(2.01,3.08)	2.72(2.19,3.37)	1.64(1.50,1.80)	<0.001
	Male	Reference	1.37(1.26,1.48)	1.58(1.46,1.71)	2.19(2.03,2.36)	1.48(1.43,1.53)	
Current smoking	No	Reference	1.52(1.37,1.68)	1.83(1.66,2.02)	2.46(2.24,2.70)	1.53(1.48,1.59)	0.07

HR (95%CI) [a]		Quartiles				Continuous	P for interaction
		Quartile 1	Quartile 2	Quartile 3	Quartile 4	Per 1 IQR Increase	
	Yes	Reference	1.32(1.17,1.49)	1.52(1.35,1.70)	2.08(1.86,2.32)	1.46(1.39,1.53)	
Current drinking	No	Reference	1.51(1.37,1.67)	1.85(1.68,2.04)	2.50(2.28,2.75)	1.55(1.49,1.61)	0.02
	Yes	Reference	1.33(1.17,1.50)	1.48(1.31,1.67)	2.02(1.80,2.27)	1.43(1.36,1.51)	
Active physical activity	No	Reference	1.46(1.33,1.59)	1.73(1.59,1.89)	2.38(2.20,2.58)	1.53(1.48,1.58)	0.12
	Yes	Reference	1.37(1.16,1.62)	1.55(1.32,1.82)	1.97(1.68,2.30)	1.41(1.32,1.52)	
Hypertension	No	Reference	1.38(1.24,1.53)	1.55(1.40,1.72)	2.15(1.95,2.38)	1.48(1.42,1.55)	0.01
	Yes	Reference	1.24(1.10,1.39)	1.40(1.25,1.56)	1.71(1.53,1.90)	1.30(1.25,1.36)	
Diabetes	No	Reference	1.39(1.28,1.50)	1.57(1.46,1.70)	2.07(1.92,2.24)	1.44(1.39,1.49)	0.02
	Yes	Reference	1.65(1.13,2.40)	1.93(1.36,2.75)	2.07(1.47,2.93)	1.23(1.14,1.33)	
Hyperlipidemia	No	Reference	1.42(1.31,1.55)	1.69(1.56,1.83)	2.27(2.10,2.45)	1.50(1.45,1.55)	0.45
	Yes	Reference	1.35(1.08,1.68)	1.48(1.20,1.83)	1.97(1.61,2.40)	1.38(1.29,1.49)	
**TyG-WHtR**							
Age group	<60 years	Reference	1.68(1.48,1.91)	2.07(1.82,2.34)	2.94(2.61,3.32)	1.68(1.60,1.77)	<0.001
	≥60 years	Reference	1.20(1.09,1.32)	1.49(1.36,1.63)	1.95(1.78,2.13)	1.42(1.37,1.47)	

HR (95%CI) [a]		Quartiles				Continuous	P for interaction
		Quartile 1	Quartile 2	Quartile 3	Quartile 4	Per 1 IQR Increase	
Gender	Female	Reference	1.51(1.16,1.98)	2.45(1.91,3.15)	3.07(2.41,3.91)	1.64(1.51,1.79)	<0.001
	Male	Reference	1.37(1.27,1.49)	1.66(1.53,1.79)	2.23(2.07,2.41)	1.50(1.45,1.55)	
Current smoking	No	Reference	1.43(1.29,1.58)	1.80(1.63,1.98)	2.44(2.22,2.68)	1.55(1.49,1.61)	0.52
	Yes	Reference	1.35(1.20,1.52)	1.66(1.48,1.86)	2.18(1.96,2.44)	1.49(1.42,1.56)	
Current drinking	No	Reference	1.46(1.32,1.62)	1.87(1.70,2.06)	2.51(2.29,2.76)	1.56(1.50,1.62)	0.14
	Yes	Reference	1.30(1.15,1.47)	1.56(1.39,1.75)	2.11(1.88,2.36)	1.47(1.40,1.54)	
Active physical activity	No	Reference	1.44(1.32,1.57)	1.77(1.63,1.93)	2.41(2.22,2.61)	1.54(1.49,1.59)	0.18
	Yes	Reference	1.22(1.03,1.45)	1.60(1.36,1.88)	2.05(1.76,2.40)	1.45(1.36,1.56)	
Hypertension	No	Reference	1.30(1.18,1.44)	1.63(1.47,1.80)	2.16(1.96,2.39)	1.50(1.44,1.57)	<0.01
	Yes	Reference	1.24(1.10,1.40)	1.42(1.26,1.58)	1.76(1.58,1.96)	1.32(1.27,1.38)	
Diabetes	No	Reference	1.36(1.26,1.47)	1.61(1.49,1.74)	2.11(1.96,2.28)	1.46(1.41,1.51)	0.01
	Yes	Reference	1.54(1.05,2.25)	2.03(1.42,2.90)	2.10(1.48,2.97)	1.27(1.18,1.37)	
Hyperlipidemia	No	Reference	1.38(1.27,1.50)	1.74(1.60,1.88)	2.30(2.13,2.48)	1.52(1.47,1.57)	0.31
	Yes	Reference	1.35(1.09,1.69)	1.49(1.21,1.84)	2.04(1.67,2.49)	1.42(1.32,1.53)	

**HR (95%CI) [a]**		**Quartiles**				**Continuous**	**P for interaction**
		**Quartile 1**	**Quartile 2**	**Quartile 3**	**Quartile 4**	**Per 1 IQR Increase**	
	Yes	Reference	1.41(1.10,1.82)	1.45(1.14,1.85)	2.06(1.64,2.59)	1.43(1.31,1.55)	

[a] Cox proportional hazard regression model, adjusted for age, gender, monthly income, education level, marital status, current smoking, current drinking, physical activity, and family history of atherosclerotic cardiovascular diseases.

Abbreviations: ASCVD, atherosclerotic cardiovascular disease; BMI, body mass index; HR (95%CI), hazard ratio (95% confidence interval); IQR, interquartile range; TyG, triglyceride-glucose; WC, waist circumference; WHtR, Waist-to-Height ratio.

Supplementary Table 6. Sensitivity analyses for association of TyG and its related parameters with atherosclerotic cardiovascular disease and its subtypes.

HR (95%CI)	Quartiles				Continuous
	Quartile 1	Quartile 2	Quartile 3	Quartile 4	Per 1 IQR Increase
**Excluding ASCVD events occurred during the initial two years of follow-up**					
**ASCVD**					
TyG	Reference	1.26 (1.16, 1.36)	1.54 (1.43, 1.66)	1.97 (1.84, 2.12)	1.39 (1.35, 1.43)
TyG-BMI	Reference	1.33 (1.24, 1.44)	1.57 (1.46, 1.69)	2.10 (1.96, 2.25)	1.45 (1.41, 1.50)
TyG-WC	Reference	1.42 (1.31, 1.53)	1.68 (1.55, 1.81)	2.28 (2.12, 2.46)	1.50 (1.45, 1.55)
TyG-WHtR	Reference	1.38 (1.27, 1.50)	1.72 (1.60, 1.86)	2.32 (2.15, 2.50)	1.51 (1.47, 1.56)
**Myocardial Infarction**					
TyG	Reference	1.32 (1.11, 1.56)	1.94 (1.65, 2.27)	2.54 (2.18, 2.96)	1.51 (1.43, 1.61)
TyG-BMI	Reference	1.30 (1.10, 1.54)	1.70 (1.45, 1.99)	2.43 (2.09, 2.82)	1.54 (1.45, 1.65)
TyG-WC	Reference	1.69 (1.41, 2.03)	2.18 (1.83, 2.60)	3.10 (2.61, 3.67)	1.64 (1.54, 1.75)
TyG-WHtR	Reference	1.57 (1.31, 1.88)	2.22 (1.87, 2.63)	2.92 (2.48, 3.45)	1.64 (1.53, 1.74)
**Ischemic Stroke**					
TyG	Reference	1.24 (1.14, 1.35)	1.44 (1.33, 1.56)	1.84 (1.70, 1.99)	1.35 (1.31, 1.39)

HR (95%CI)	Quartiles				Continuous
	**Quartile 1**	**Quartile 2**	**Quartile 3**	**Quartile 4**	**Per 1 IQR Increase**
TyG-BMI	Reference	1.33 (1.23, 1.45)	1.53 (1.41, 1.66)	2.00 (1.85, 2.17)	1.42 (1.37, 1.47)
TyG-WC	Reference	1.35 (1.24, 1.47)	1.56 (1.43, 1.70)	2.11 (1.94, 2.29)	1.46 (1.41, 1.51)
TyG-WHtR	Reference	1.32 (1.21, 1.45)	1.61 (1.48, 1.75)	2.17 (2.00, 2.36)	1.48 (1.43, 1.53)
**Using the updated mean instead of baseline measurements of TyG index and its related parameters**					
**ASCVD**					
TyG	Reference	1.12 (1.04, 1.20)	1.27 (1.18, 1.36)	1.53 (1.42, 1.64)	1.25 (1.21, 1.29)
TyG-BMI	Reference	1.18 (1.10, 1.27)	1.35 (1.26, 1.45)	1.63 (1.52, 1.75)	1.29 (1.25, 1.33)
TyG-WC	Reference	1.25 (1.16, 1.35)	1.41 (1.31, 1.52)	1.79 (1.66, 1.92)	1.35 (1.31, 1.39)
TyG-WHtR	Reference	1.24 (1.14, 1.33)	1.48 (1.38, 1.60)	1.85 (1.72, 1.99)	1.39 (1.35, 1.43)
**Myocardial Infarction**					
TyG	Reference	1.19 (1.02, 1.39)	1.44 (1.24, 1.67)	1.86 (1.60, 2.15)	1.33 (1.26, 1.42)
TyG-BMI	Reference	1.10 (0.94, 1.28)	1.42 (1.22, 1.64)	1.73 (1.50, 2.00)	1.34 (1.26, 1.43)
TyG-WC	Reference	1.50 (1.27, 1.77)	1.56 (1.33, 1.84)	2.28 (1.94, 2.67)	1.43 (1.34, 1.52)
TyG-WHtR	Reference	1.36 (1.15, 1.60)	1.64 (1.40, 1.92)	2.21 (1.89, 2.58)	1.45 (1.37, 1.54)
**Ischemic Stroke**					

HR (95%CI)	Quartiles				Continuous
	Quartile 1	Quartile 2	Quartile 3	Quartile 4	Per 1 IQR Increase
TyG	Reference	1.09 (1.01, 1.18)	1.21 (1.12, 1.31)	1.44 (1.33, 1.55)	1.22 (1.18, 1.26)
TyG-BMI	Reference	1.19 (1.10, 1.29)	1.33 (1.23, 1.44)	1.58 (1.46, 1.71)	1.26 (1.22, 1.31)
TyG-WC	Reference	1.18 (1.09, 1.29)	1.35 (1.24, 1.47)	1.67 (1.54, 1.81)	1.32 (1.27, 1.37)
TyG-WHtR	Reference	1.19 (1.09, 1.30)	1.43 (1.31, 1.55)	1.74 (1.61, 1.89)	1.36 (1.32, 1.41)
**Further adjusting for hypertension, diabetes and hyperlipidemia**					
**ASCVD**					
TyG	Reference	1.21 (1.12, 1.30)	1.34 (1.25, 1.44)	1.51 (1.40, 1.62)	1.21 (1.17, 1.25)
TyG-BMI	Reference	1.23 (1.14, 1.32)	1.36 (1.26, 1.46)	1.57 (1.46, 1.69)	1.24 (1.20, 1.28)
TyG-WC	Reference	1.32 (1.22, 1.42)	1.46 (1.35, 1.57)	1.75 (1.62, 1.88)	1.30 (1.26, 1.34)
TyG-WHtR	Reference	1.29 (1.19, 1.39)	1.49 (1.39, 1.61)	1.79 (1.66, 1.92)	1.32 (1.28, 1.36)
**Myocardial Infarction**					
TyG	Reference	1.27 (1.08, 1.50)	1.62 (1.39, 1.89)	1.94 (1.67, 2.27)	1.31 (1.23, 1.40)
TyG-BMI	Reference	1.20 (1.02, 1.40)	1.42 (1.22, 1.65)	1.81 (1.56, 2.10)	1.31 (1.23, 1.40)
TyG-WC	Reference	1.54 (1.29, 1.83)	1.88 (1.59, 2.22)	2.35 (1.99, 2.77)	1.42 (1.33, 1.52)
TyG-WHtR	Reference	1.41 (1.19, 1.67)	1.83 (1.55, 2.15)	2.17 (1.84, 2.55)	1.41 (1.32, 1.51)

HR (95%CI)	Quartiles				Continuous
	**Quartile 1**	**Quartile 2**	**Quartile 3**	**Quartile 4**	**Per 1 IQR Increase**
**Ischemic Stroke**					
TyG	Reference	1.19 (1.10, 1.29)	1.26 (1.16, 1.36)	1.40 (1.29, 1.51)	1.17 (1.13, 1.21)
TyG-BMI	Reference	1.23 (1.14, 1.34)	1.33 (1.23, 1.44)	1.50 (1.38, 1.63)	1.21 (1.17, 1.26)
TyG-WC	Reference	1.27 (1.16, 1.38)	1.35 (1.24, 1.47)	1.62 (1.49, 1.76)	1.26 (1.21, 1.31)
TyG-WHtR	Reference	1.24 (1.14, 1.35)	1.41 (1.29, 1.53)	1.68 (1.55, 1.82)	1.29 (1.24, 1.33)
**Adopting the Cox regression model with time-varying variables updated in each survey before the occurrence of ASCVD events**					
**ASCVD**					
TyG	Reference	1.30 (1.08, 1.56)	1.58 (1.33, 1.88)	2.00 (1.69, 2.36)	1.39 (1.31, 1.49)
TyG-BMI	Reference	1.31 (1.09, 1.57)	1.75 (1.47, 2.08)	1.98 (1.67, 2.35)	1.42 (1.33, 1.52)
TyG-WC	Reference	1.67 (1.38, 2.01)	1.69 (1.40, 2.04)	2.21 (1.85, 2.65)	1.45 (1.35, 1.56)
TyG-WHtR	Reference	1.48 (1.23, 1.79)	1.76 (1.47, 2.11)	2.08 (1.74, 2.48)	1.44 (1.34, 1.54)
**Myocardial Infarction**					
TyG	Reference	1.31 (0.91, 1.87)	1.50 (1.06, 2.13)	2.30 (1.66, 3.18)	1.45 (1.28, 1.65)
TyG-BMI	Reference	1.42 (1.00, 2.02)	1.68 (1.19, 2.36)	2.07 (1.48, 2.88)	1.43 (1.25, 1.63)
TyG-WC	Reference	1.71 (1.17, 2.49)	1.92 (1.33, 2.78)	2.26 (1.58, 3.24)	1.51 (1.32, 1.72)

HR (95%CI)	Quartiles				Continuous
	**Quartile 1**	**Quartile 2**	**Quartile 3**	**Quartile 4**	**Per 1 IQR Increase**
TyG-WHtR	Reference	1.50 (1.04, 2.17)	1.89 (1.33, 2.69)	2.07 (1.46, 2.93)	1.48 (1.30, 1.69)
**Ischemic Stroke**					
TyG	Reference	1.30 (1.05, 1.60)	1.59 (1.30, 1.94)	1.93 (1.59, 2.34)	1.39 (1.29, 1.49)
TyG-BMI	Reference	1.25 (1.01, 1.54)	1.77 (1.45, 2.15)	1.96 (1.61, 2.38)	1.42 (1.32, 1.54)
TyG-WC	Reference	1.64 (1.32, 2.03)	1.58 (1.28, 1.96)	2.22 (1.81, 2.72)	1.45 (1.33, 1.57)
TyG-WHtR	Reference	1.49 (1.20, 1.85)	1.71 (1.39, 2.11)	2.11 (1.72, 2.58)	1.44 (1.33, 1.55)
**Adopting the Fine & Gary sub-distribution hazard models to further consider the competing risk of non-ASCVD deaths**					
**ASCVD**					
TyG	Reference	1.30 (1.21, 1.40)	1.55 (1.44, 1.66)	2.00 (1.87, 2.14)	1.38 (1.35, 1.42)
TyG-BMI	Reference	1.37 (1.27, 1.48)	1.64 (1.53, 1.76)	2.16 (2.01, 2.31)	1.46 (1.42, 1.50)
TyG-WC	Reference	1.45 (1.35, 1.57)	1.70 (1.58, 1.84)	2.28 (2.12, 2.45)	1.49 (1.44, 1.53)
TyG-WHtR	Reference	1.40 (1.30, 1.51)	1.73 (1.61, 1.87)	2.29 (2.13, 2.46)	1.50 (1.46, 1.54)
**Myocardial Infarction**					
TyG	Reference	1.36 (1.16, 1.60)	1.86 (1.60, 2.16)	2.55 (2.21, 2.95)	1.50 (1.43, 1.58)
TyG-BMI	Reference	1.32 (1.13, 1.54)	1.70 (1.47, 1.98)	2.46 (2.14, 2.83)	1.55 (1.46, 1.64)

HR (95%CI)	Quartiles				Continuous
	Quartile 1	Quartile 2	Quartile 3	Quartile 4	Per 1 IQR Increase
TyG-WC	Reference	1.67 (1.41, 1.99)	2.16 (1.82, 2.55)	3.03 (2.58, 3.56)	1.63 (1.54, 1.72)
TyG-WHtR	Reference	1.52 (1.28, 1.80)	2.09 (1.78, 2.46)	2.78 (2.38, 3.25)	1.61 (1.52, 1.71)
**Ischemic Stroke**					
TyG	Reference	1.28 (1.19, 1.39)	1.46 (1.35, 1.57)	1.86 (1.72, 2.00)	1.35 (1.31, 1.39)
TyG-BMI	Reference	1.38 (1.27, 1.50)	1.61 (1.49, 1.75)	2.07 (1.92, 2.23)	1.43 (1.38, 1.48)
TyG-WC	Reference	1.40 (1.29, 1.53)	1.58 (1.46, 1.72)	2.12 (1.96, 2.30)	1.45 (1.40, 1.50)
TyG-WHtR	Reference	1.36 (1.25, 1.48)	1.63 (1.51, 1.78)	2.16 (1.99, 2.34)	1.46 (1.41, 1.51)

Abbreviations: ASCVD, atherosclerotic cardiovascular disease; BMI, body mass index; HR (95%CI), hazard ratio (95% confidence interval); IQR, interquartile range; TyG, triglyceride-glucose; WC, waist circumference; WHtR, Waist-to-Height ratio.

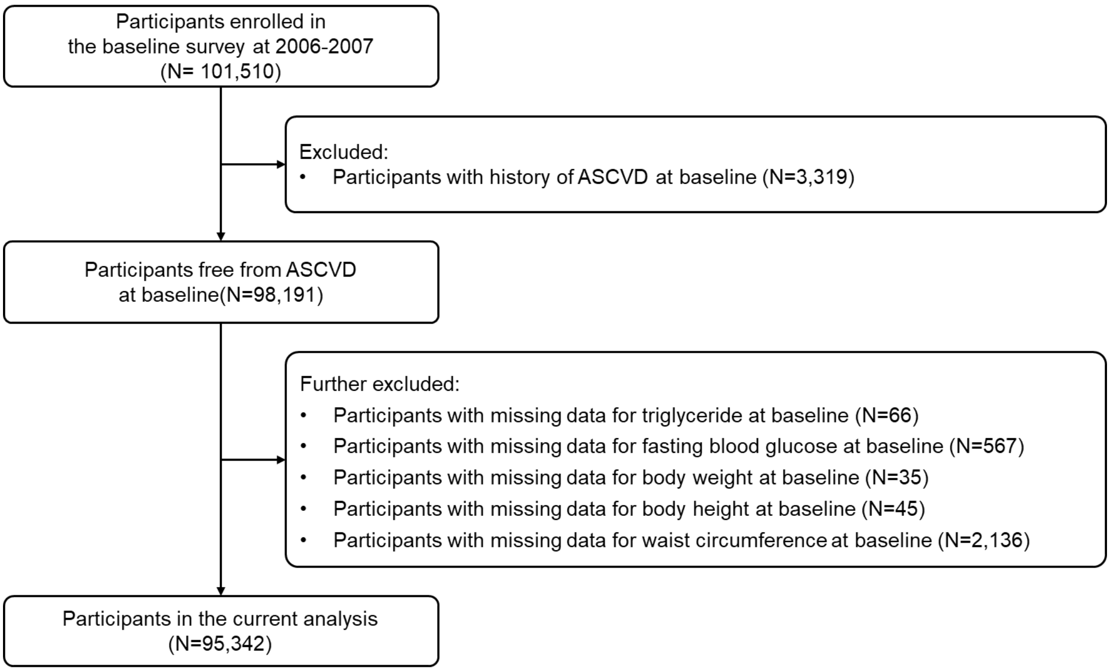

Supplementary Figure 1. Flow chart of participants included and excluded in the analyses.

Abbreviations: ASCVD, atherosclerotic cardiovascular disease.

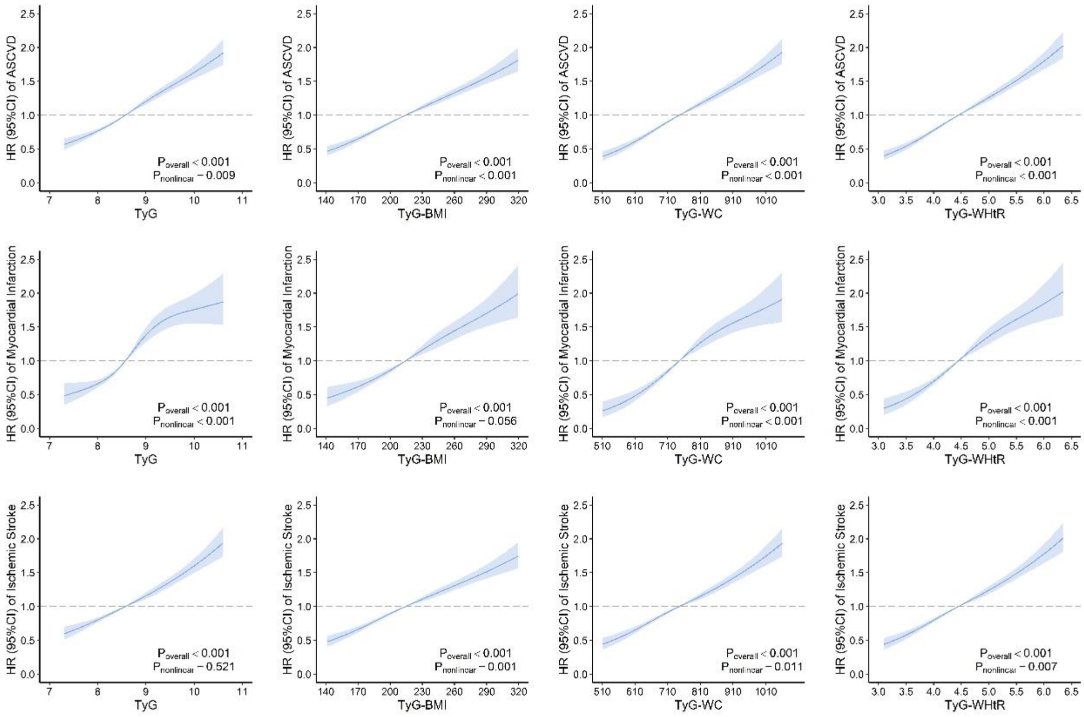

Supplementary Figure 2. Restricted cubic spline curves of the association of triglyceride-glucose and its related parameters with atherosclerotic cardiovascular disease and its subtypes.

Abbreviations: ASCVD, atherosclerotic cardiovascular disease; BMI, body mass index; HR (95%CI), hazard ratio (95% confidence interval); IQR, interquartile range; TyG, triglyceride-glucose; WC, waist circumference; WHtR, Waist-to-Height ratio.
